# Evaluation of Ambulatory Geriatric Rehabilitation (EAGER): study protocol of a matched cohort study based on claims data

**DOI:** 10.1186/s12877-017-0452-1

**Published:** 2017-03-02

**Authors:** Simone Kiel, Carolin Zimak, Jean-François Chenot, Carsten Oliver Schmidt

**Affiliations:** 1 Department of General Practice, Institute of Community Medicine, University Medicine Greifswald, Greifswald, Germany; 2 Department of SHIP-KEF, Institute of Community Medicine, University Medicine Greifswald, Greifswald, Germany; 3 Institut für Community Medicine—Abteilung Allgemeinmedizin, Universitätsmedizin Greifswald, KdöR, Fleischmannstraße 42, 17475 Greifswald, Germany

**Keywords:** Ambulatory geriatric rehabilitation, Geriatric multimorbidity, Health claims data, Matched cohort study, Propensity score matching

## Abstract

**Background:**

Ambulatory geriatric rehabilitation (AGR) is a community based outpatient intervention which aims to improve physical function, maintain independent living of geriatric patients, avoiding hospitalisation and institutionalisation. It should therefore reduce health care costs. The objective of our study is to evaluate the effectiveness of AGR for frail elderly patients insured by the statutory health insurance AOK Nordost compared to care as usual. Outcome variables are progression to higher nursing care levels, admission to nursing home, incident fractures, hospital admission, ambulatory care sensitive hospital admissions, days spent in hospital, and health care costs.

**Methods:**

This matched cohort study aims to estimate average treatment effects of AGR. For this purpose we will compare patients receiving AGR with matched patients receiving care as usual. Patients in the intervention group were treated between the years 2009 and 2013 from three centres in Mecklenburg-Vorpommern, Germany. Matching will be conducted using propensity score techniques. Claims data will be provided by the statutory health insurance AOK Nordost. The primary outcomes are the progression of nursing care levels, incident fractures, and admission to nursing home. Secondary outcomes are total and ambulatory care sensitive hospital admissions, and health care costs from the statutory health insurance perspective. Data will be analysed using appropriate regression models.

**Discussion:**

This study aims to quantify the effectiveness of AGR. Results will be important for providers of AGR, policy makers and stakeholders to make informed decisions on whether to continue, modify or extend AGR.

**Trial registration:**

German Clinical Trials Register (DRKS) S00008926, registered 29.07.2015

## Background

Many elderly will experience a reduction in physical function, leading to more falls and injuries. This leads to a loss of independence, hospitalisation, long-term nursing home care as well as premature death [1, 2]. In 2013, 2.63 million people in Germany were in need of nursing care. Roughly two third of them received ambulatory nursing care and one third received care in a nursing home. From the years 2011 to 2013, people receiving nursing care increased by 5% [3]. In 2014, 27% of the population aged 65–79 received either ambulatory or institutionalised nursing care [4]. Given the projected demographic changes in Germany, the population in the age group ≥65 years will increase from 21% (17.3 million) in the year 2015 to 27% (21.8 million) in the year 2030 [5]. Long term nursing care and institutionalisation in a nursing home can be associated with a significant reduction of quality of life, mainly due to loss of autonomy and social contacts [6]. The increased need of long term nursing care poses a major economic challenge [7]. Thus measures to prevent, minimise or delay long term nursing care for elderly are urgently needed.

Germany has mandatory nursing care insurance attached to statutory health insurance since 1995. Elderly with disabilities or dementia can apply for a nursing care level [*Pflegestufe*]. To identify elderly in need, a basic geriatric assessment performed in general practice was introduced in 2005 [8]. Although elderly are entitled to rehabilitative services as codified in the Book V (SGB V) and XI (SGB XI) of the German social code general practitioners have only limited access to specialised rehabilitative services for geriatric patients. While the number of hospitals providing geriatric services is increasing, the demand for ambulatory services is not met [9]. Elderly patients with a need for rehabilitation prefer to stay close to their home and relatives, maintaining their everyday life [10]. Consequently, ambulatory rehabilitation is preferred. Rehabilitative services were and are still mainly available after hospitalisation e.g. for stroke or fracture after a fall.

Preventive ambulatory geriatric rehabilitation (AGR [*Ambulante Geriatrische Komplexbehandlung*]) was introduced in 2008 within the legal frame (§ 140 Book V of the social code) of selective contracts for integrated care. Therefore it is only available as a model intervention in some areas for holders of specific statutory health insurances (e.g. AOK Nordost). AGR is not part of regular health care.

It is intended as a community based outpatient intervention to improve patient’s physical function, increase patient’s safety and quality of life as well as to prevent falls and injuries, to avoid and delay hospitalisation, the progression of nursing care level and admission to nursing home.

A systematic review of controlled trials of ambulatory and hospital interventions to improve physical function and maintain independent living in elderly people concluded that they were effective to achieve the goal [1]. This review comprised only one German trial which included geriatric patient post hospital discharge [11]. However, AGR is primarily intended to prevent hospitalisation and not as a post-discharge rehabilitation. The effectiveness of German AGR programs has not been evaluated rigorously yet. Previous studies have relied on uncontrolled study designs [12].

### Objectives and hypotheses

The aim of our study is to evaluate the effectiveness of AGR regarding patient’s progression to higher nursing care levels, incident fractures, admission to nursing home, hospital admissions as well as health care costs. For this purpose we compare patients receiving AGR with patients receiving care as usual. We will estimate average treatment effects based on a cohort design using propensity score techniques to match cases and controls. The follow-up period will be up to 2 years.

*Our primary hypotheses are*:

1. AGR reduces and delays progression to higher nursing care levels.
2. AGR reduces and delays nursing home admissions.
3. AGR reduces the risk of incident fractures.

*Our secondary hypotheses are*:

1. AGR decreases and delays hospital admissions and reduces the days spent in hospital during follow-up time.
2. AGR decreases and delays ambulatory care sensitive hospital admissions.
3. AGR decreases total health care costs from the statutory health insurance perspective.

On an exploratory basis we will investigate the effect on drug prescriptions.

## Methods

### Study design

The conduct of a randomised controlled trial to assess the effectiveness of AGR is currently limited due to logistic and ethical reasons. Therefore we will conduct a matched cohort study using claims data. Anonymised data will be provided by the statutory health insurance AOK Nordost which comprises basic demographic data, data on nursing care level, admission to a nursing home, billing data for ambulatory services (EBM position numbers) and for hospital services (DRG-codes/OPS-codes), as well as diagnoses (ICD-10 codes), and all health care costs, including costs for hospitalisation, remedies and aids, ambulatory costs and costs for medication. To balance potential confounders, we will apply a propensity score matching. Controls will be matched patients insured by the AOK Nordost.

The observation period comprises 4 billing periods (each 3 months, corresponding to 1 year) prior to the intervention, the intervention (index) billing period, and up to 8 billing periods post intervention, resulting in a total observation period of up to 13 billing periods. The billing period covering the most days of the intervention will be considered as the index billing period. Participants in the intervention group received a 4 weeks AGR in between the years 2009 and 2013.

### Description of the AGR intervention and the setting

**Fig. 1:**
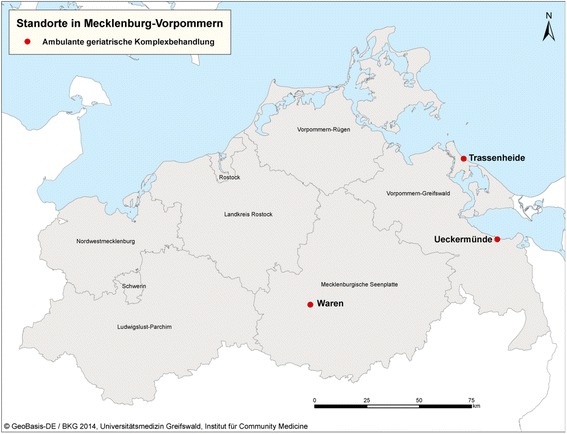
Map of Mecklenburg-Vorpommern with the three locations which provided AGR

AGR is a multimodal intervention consisting of physiotherapy, ergotherapy, speech therapy, occupational therapy, social support by qualified social workers, psychological counselling and counselling regarding aids and care. Patients who are deemed suitable for AGR by their general practitioners can be referred to special rehabilitation centre for a geriatric assessment. If they fulfil eligibility criteria for AGR and agree to participate they receive the intervention. The intervention is tailored to the patients’ needs and delivered in individual and group sessions. Patients are commonly treated for a total of 20 days with two to three 30 min therapy units per day. Included are meals and a pick up and return service for the elderly every day. During the intervention period AGR was available in three locations in Mecklenburg-Vorpommern (Trassenheide, Ueckermünde, Waren) (Fig. 1).

### Eligibility criteria for AGR

Due to selective contracts only patients insured by the AOK Nordost are entitled to receive AGR. The geriatric assessment comprises the activity of daily living scale (Barthel-Index), instrumental activity of daily living, Timed “Up & Go”, Chair-Rising-Test, Tandem Stance, Berg-Balance-Scale, and handgrip strength. Eligibility criteria are:

1. Aged 70 and older
2. at least two geriatric multimorbidity listed in Table 1
3. impairment or handicap with functional deficits
4. at least one of the health conditions listed in Table 2

**Table 1:** Geriatric multimorbidity

Criteria	ICD-10 codes
Immobility	M96.8, M62.3, M62.5
cognitive impairment	G30, F00 – F07
incontinence	R32, N39, R15
decubitus ulcer	L89, L97, I83, L98
Malnutrition	R64, E41, E43, E44
Disturbances in fluid and electrolyte balance	E86, E87, R60
Depression and Anxiety	F30 – F33, F40, F41
chronic pain	R52
paraesthesia	R20, G50 – G59, G60 – G64
frailty	R54
severe visual or hearing impairment	H25, H28, H52-H54, H90, H91

**Table 2:** Eligibility criteria for participation in AGR

Eligibility criteria	ICD-10 codes
Stroke and other cerebrovascular disorders	I60-I69
Status post fracture	S72.-
Arterial obstructive disease with amputation or other surgery	I70.-
Cox and osteoarthritis with Implantation of an endoprosthesis	M16.-, M17.-
Heart failure	I50.-
Exacerbated chronic obstructive pulmonary disease	J44.0, J44.1
Pneumonia and other respiratory tract infections	J10-J22
Other fractures and injuries	S00-T98
Other arthropathies	M00-M25
Spondylopathies and Discopathies, possibly with laminectomy	M45-M51
Coronary heart disease and other heart diseases with cardiac surgery	I05-I09; I20-I25
Delirium and other organic brain psychosis	F00-F05
Osteoporosis	M80-M81
Secondary Parkinsonsyndrom	G20.9
Symptoms, effecting the nervous system and musculoskeletal system	R29.5, R29.6, R29.8

Patients are not eligible for AGR if the assessment indicates a need for hospital admission, if they are not able to participate due to a poor health status or if they are unable to provide informed consent.

### Exclusion criteria from the analyses

We aim to quantify treatment effects for typical AGR patients. Therefore, we will exclude participants with rare conditions which are likely to affect the health course beyond the effects which can be reasonably assumed from AGR (e.g. organ transplantation, dialysis, chemotherapy) or participants with extremely high health care costs indicating severe conditions. Prespecified criteria used to exclude AGR participants from our analyses are shown in Table 3. Exclusion criteria for AGR participants also apply to potential controls.

**Table 3:** Criteria for the exclusion of AGR participants for study analysis

Exclusion criteria	
- Age <70 or >95 years in the intervention billing period Prior to the intervention billing period: - nursing care >2 - <360 insured days in the four previous billing periods - living in a nursing home - hospital costs without out of pocket spending at the last billing period >33.000 € - hospital costs without out of pocket spending during the four last billing periods ≥44.000 € - ambulatory costs during the last billing period ≥2.200 € - ambulatory costs during the four last billing periods ∑ ≥5.500€ - remedy costs without out of pocket spending during the four previous billing periods ≥2.200 € - costs of aid without out of pocket spending during the four previous billing periods ≥4.000 € - drug costs without out of pocket spending during the four previous billing periods AGR ≥11.000 € - total health care costs without out of pocket spending during the four previous billing periods ≥44.000 € - diagnosed AIDS/HIV in the four previous billing periods - chemotherapy in the four previous billing periods - organ transplantation during the four previous billing periods - dialysis during the four previous billing periods	

### Sample size

The intervention group consists of all AGR participants, receiving the AGR in Mecklenburg-Vorpommern during the years 2009–2013. This will comprise approximately 700 patients. Controls will be chosen from a pool of around 250.000 members of the AOK Nordost aged 70 years and older. Approximately 2800 controls will be selected from the pool.

### Outcome measures and data collection

Claims data will comprise the billing periods from 01.01.2008 until 31.12.2014, thus ensuring a minimal observation period of 12 months prior and after the index billing period. All variables are provided for each billing period during the observation period.

The primary outcomes are ‘*progression of nursing care level*’, ‘*admission to nursing home*’ and ‘*incident fractures*’.

Four nursing care levels (0–3) are defined by the German social code XI (SGB XI). The nursing care level needs to be approved by the Medical Review Board of the statutory health insurances (MDK) in a standardised procedure. The definition for nursing care level is described in Table 4 [13]. Elderly who do not reach the requirements for nursing care level I, but need help for daily living (SGB XI § 45a) receive care for their needs. This is referred to as nursing care level 0 [13].

**Table 4:** Definitions of the nursing care levels [13]

	Requirements	
Nursing care level	total daily help (including help in household)	personal help (included in total daily help)
1	minimum 1,5 h	>45 min
2	minimum 3 h	≥2 h, 3 times a day
3	minimum 5 h	≥4 h permanent help

The outcomes ‘*admission to nursing home*’ and ‘*incident fractures*’ are both coded as binary variables, stating whether the patients experienced the event after the index billing period or not.

Secondary outcome variables are ‘any *hospital admission*’ (yes/no), ‘*days spent in the hospital*’, ‘*ambulatory care sensitive hospital admissions*’ (yes/no) and ‘*total health care costs from the statutory health insurance perspective*’ during the entire follow-up period and during each billing period in the follow-up period. Ambulatory care sensitive hospital admissions are defined as potentially preventable hospital admissions by interventions in primary care and are displayed in Table 5. Conditions were chosen from two studies [14, 15].

**Table 5:** Ambulatory care sensitive conditions used to define hospital admissions to be prevented by AGR (according to [14, 15])

Ambulatory care sensitive conditions	ICD-10 code
Ischaemic heart diseases	I25.0, I25.1, I25.5, I25.6, I25.8, I25.9
Heart failure	I50
Other diseases of the circulatory system	I05, I06, I08.0, I49.8, I49.9, I67.2, I67.4, I70, I73, I78, I80.0, I80.80, I83, I86, I87, I95, R00.0, R00.2
Bronchitis & COPD	J20, J21, J40-J44, J47
Mental and behavioural disorders due to use of alcohol or opioids	F10, F11
Back pain [dorsopathies]	M42, M47, M53, M54
Hypertension	I10 - I15
Gastroenteritis and other diseases of intestines	K52.2, K52.8, K52.9, K57, K58, K59.0
Intestinal infectious diseases	A01, A02, A04, A05, A07-A09
Influenza and pneumonia	J10, J11, J13, J14, J15.3, J15.4, J15.7, J15.8, J15.9, J16.8, J18.0, J18.1, J18.8, J18.9
Ear nose throat infections	H66, H67, J01-J04, J06, J31, J32, J35
Depressive disorders	F32, F33
Diabetes mellitus	E10.2-E10.6, E10.8, E10.9, E11, E13.6, E13.7, E13.9, E14, E16.2
Gonarthrosis [arthrosis of knee]	M17.0, M17.1, M17.4, M17.5, M17.9
Soft tissue disorders	G56.0, M67.4, M71.3, M75-M77, M79
Other avoidable mental and behavioural disorders	F40, F41, F43, F45, F50.0, F50.2, F60
Diseases of the eye	H25, H40
Diseases of urinary system	N30, N34, N39.0
Sleep disorders	G47
Diseases of the skin and subcutaneous tissue	A46, L01, L02, L04, L08.0, L08.8, L08.9, L60.0, L72.1, L98.0
Thyroid disorder	E03 - E05, E89.0
Metabolic disorders	E86, E87.6
Melanoma and other malignant neoplasms of skin	C43, C44
Gastritis and duodenitis	K21, K29.7, K29.9, K30, K31
Migraine and headache syndromes	G43, G44.0, G44.1, G44.3, G44.4, G44.8, R51
Malnutrition & nutritional deficiencies	E40 - 64, R63.6, D50, D51-D52, D53.1, D56
Alcoholic liver disease	K70
Dental diseases	K02, K04-K06, K08, K12, K13
Inflammatory diseases of female pelvic organs and disorders of female genital tract	N70-N72, N75, N76, N84.1, N86, N87
Dementia	F01, F03
Diseases of male genital organs	N41, N45, N48.4
Asthma	J45
Other polyneuropathies	G62
Avoidable infectious and parasitic diseases	A15.3, A15.4, A15.9, A16.2, A16.3, A16.5, A16.8, A16.9, A34 - A37, A50-A58, A63, A64, A80, B05 - B07, B15, B16.1, B16.9, B17, B18.0, B18.1, B20 - B24, B26, B34.9, B51 - B54, B77, B86
Convulsions, not elsewhere classified	R56
Decubitus ulcer and pressure area	L89
Obesity	E66
Rare diseases with <5000 cases each	F80, R63.0, R63.3, R63.8, Z73
Cellulitis	L03, L04, L08.0, L08.8, L08.9, L88, L98.0, I891, L010, L011, L020 - L024, L028, L029
Angina	I20, I24.0, I24.8, I24.9, I25, R072, R073, R074, Z034, Z035
Convulsions and epilepsy	G25.3, G40, G41, O15, R56, R568
Dehydration and infections	A02, A04, A09, A05.9, A07.2, A08.0, A08.1, A08.3, A08.4, A08.5, E86, K52.0, K52.1, K52.2, K52.8, K52.9
Gangrene	R02
Iron-deficiency anaemia	D46.0, D46.1, D46.3, D46.4, D50.1, D50.8, D50.9, D51.0–D51.3, D51.8, D52.0, D52.1, D52.8, D52.9, D53.1, D57.1, D58.0, D58.1, D59.0 – D59.2, D59.9, D60.1, D60.8, D60.9, D61.0, D61.1, D64.0 - D64.4, D64.8
Pelvic inflammatory disease	N70, N73, N74
Perforated/bleeding ulcer	K20, K21.0, K21.9, K22.1, K22.6, K25.0 – K25.2, K25.4 – K25.6, K25.9, K26.0 – K26.2, K26.4 – K26.6, K27,K28.0 – 28.2, K28.4 – K28.6, K92.0, K92.1, K92.2, L97
Pyelonephritis	N10, N11, N12, N13.6, N15.9, N30.0, N30.8, N30.9, N39.0,
Atrial fibrillation and flutter	I47.1, I47.9, I49.5, I49.8, I49.9, R00.0, R00.2, R00.8
Constipation	K59.0
Deliberate self-harm	S16
Dyspepsia and other stomach function disorders	K21, K30
Hypokalaemia	E87.6
Neuroses	E10, E13.6 – E13.9, E14.9
Tuberculosis	A15, A16, A17, A18, A19
Schizophrenia	F20, F21, F23.2, F25
Stroke	I61, I62, I63, I64, I66, I67.2, I69.8, R47.0

‘*Total health care costs*’ comprises expenditures for hospitalisation, remedies and medical aids, ambulatory costs, and medication. The variables concerning costs refer to costs excluding out-of-pocket spending except for remedies and medical aids where the available data does not allow for any separate analysis of out-of-pocket spending.

### Matching and statistical analyses

To balance the distribution of potential confounders among cases and controls we will conduct a propensity score matching using a many-to-one matching [16]. Variables for the estimation of the propensity score will be selected based on their expected importance to predict AGR participation as well as the outcomes of interest.

We will perform a two-step matching process. In the first step, for all patients who received AGR in a determined billing period, we plan to match controls with similar morbidity and cost characteristics during a.) the four billing periods prior to the index period in which the intervention took place, and b.) by additionally including the index period in the matching. This first step allows for the definition of an index period in controls. Using variables during the index billing period is complicated by the fact that no information on the temporal sequence of events within a billing period is available due to legally obliged data protection agreements. However, ignoring the index period may lead to a systematic ignorance of events leading to AGR which might have taken place in the index period. Therefore results from both analysis scenarios (a., b.) will be systematically compared. The first-step will be conducted as an exact match (pre matching) on selected variables listed in Table 6. These variables were selected due to their high expected conceptual importance for the estimation of treatment effects. Should no adequate numbers of controls be found, a more lenient matching may be employed. Controls will be drawn with repetition across billing periods. We plan to match up to 100 controls per case at this stage.

**Table 6:** Matching criteria

Matching Criterion	Pre Matching	Propensity Score
Age	x ± 2 years	x
Sex	x	x
Area of residence		x
Days being insured		x
Level of nursing care	x	x
Hospital admission yes/no	x	x
Charlson Comorbidity index (CCI)		x
Days spent in the hospital		x
Hospital costs without out-of-pocket spending	x	x
Ambulatory costs		x
Costs of remedy		x
Costs of medical aid		x
Drug costs without out-of-pocket spending	x	x
Total costs with out-of-pocket spending		x
Main diagnoses before AGR		
*Musculo*-*skeletal*	x ^a^	
Status post fracture or joint replacement		
Cox- or gonarthrosis with endoprothesis		x
Other fractures and injuries		x
Other arthropaties		x
Osteoporosis		x
Spondylopathies and Discopathies, possibly with laminectomy		x
*Infection*		
Pneumonia and other lung inflammations	x ^a^	x
*Cardio*-*vascular system*		
Heart failure		
Chronic obstructive pulmonary disease (COPD)		x
Arterial obstructive disease with amputation or other surgery	x ^a^	x
Coronary heart diseases with surgery		x
Stroke and other cerebrovascular diseases		x
*Neuro*-*psychiatric*		
Delirium and other organic brain psychosis		x
Secondary Parkinsonsyndrom	x ^a^	x
Symptoms, effecting the nervous system and musculoskeletal system		x
Charlson Comorbidity Index		
Any Malignancy	x	x
Cerebrovascular disease		x
Chronic pulmonary disease		x
Congestive heart failure		x
Metastatic solid tumor	x	
Dementia	x	x
Hemiplegia or paraplegia		x
Mild liver disease		x
Myocardial infarction		x
Renal disease		x
Peripheral vascular disease		x
Geriatric Multimorbidity		
Cognitive deficit		x
Chronic pain		x
Depression, Anxiety		x
Incontinence		x
Severe visual/hearing impairment		x
Paraesthesia		x
Immobility	x	x
Chemotherapy	x	x

^a^At least one diagnosis from each main diagnosis group will be used to match controls. Diagnoses are allowed to differ between cases and controls within the main diagnoses group

Subsequently, propensity scores will be calculated using a logistic regression model using coded morbidity and costs under the scenarios a.) and b.), based on all variables listed in Table 6, taking statistical interactions and nonlinear associations into account. Up to four controls may be assigned to one case without repetition.

Appropriate regression models (e.g. time-event models, mixed models, two-part models) will be applied to study effects on our primary and secondary outcomes. Because treatment might also affect censoring due to mortality, competing risks models (using the Fine-Gray approach) will be applied [17]. Patients dying during the follow-up period will not be excluded from the analyses.

## Discussion

AGR is currently only available in few areas for elderly people from selected statutory health insurances which opted to offer AGR to their beneficiaries. Evaluation of this intervention was previously limited to uncontrolled study designs [12]. To the best of our knowledge, this will be the first study to evaluate AGR using a quasi-experimental design. This study may provide an estimation of the effectiveness of AGR on progression to higher nursing care levels and hospitalisation and other endpoints of clinical relevance. Our results will be important for providers of AGR, policy makers and stakeholders to make informed decisions on whether to continue, modify or even expand AGR to other areas in Germany. Additionally, our results might help to optimise AGR by identifying subgroups of patients who are more likely to benefit from AGR.

A limitation will be the restriction to claims data. Clinical measurements from instruments used for the geriatric assessment are not available for the control group. This might impair the quality of the matching as potential imbalance between clinical data and individual motivation to participate in AGR cannot be ruled out even if a high balance on claims data is achieved. Therefore, residual confounding may still be an issue after our matching. Final decisions on the applied methods need to account for the precise properties of the data.

## Abbreviations

AGR: Ambulatory geriatric rehabilitation
CCI: Charlson comorbidity index
DRG-codes/OPS-codes: German Diagnosis related Groups/Operationen- und Prozedurenschlüssel, billing data for hospital services
EBM position numbers: Uniform assessment standard, billing data for ambulatory Services (Einheitlicher Bewertungsmaßstab)
ICD-10 code: International Classification of Diseases, 10th Revision
SGB: German Social Code (Sozialgesetzbuch)

## References

[1] Beswick AD, Rees K, Dieppe P, Ayis S, Gooberman-Hill R, Horwood J, Ebrahim S (2008). Complex interventions to improve physical function and maintain independent living in elderly people: A systematic review and meta-analysis. Lancet.

[2] Hauer K, Rost B, Rütschle K, Opitz H, Specht N, Bärtsch P (2001). Exercise training for rehabilitation and secondary prevention of falls in geriatric patients with a history of injurious falls. J Am Geriatr Soc.

[3] Statistisches Bundesamt [Federal Statistical Office]. 71% der Pflegebedürftigen werden zu Hause versorgt [71% of people receive care at home]. 12.03.2015. https://www.destatis.de/DE/PresseService/Presse/Pressemitteilungen/2015/03/PD15_094_224pdf.pdf;jsessionid=0DEE7AA287E3B5653FE82021CB6F4406.cae2?__blob=publicationFile. Accessed 7 Mar 2016.

[4] Bundesministerium für Gesundheit [Federal Ministry of Health]. Zahlen und Fakten zur Pflegeversicherung, Leistungsempfaenger-nach-Altersgruppen-und-Pflegestufen-insgesamt.xls [numbers and fact of long term care insurance, recipient of benefits - by age groups and nursing care levels]. 03.05.2016. http://www.bmg.bund.de/themen/pflege/zahlen-und-fakten-zur-pflegeversicherung.html. Accessed 7 Mar 2016.

[5] Statistisches Bundesamt [Federal Statistical Office]. 13th coordinated Population Projection for Germany. 2015. file:///Z:/13.%20koordinierte%20Bev%C3%B6lkerungsvorausberechnung.htm. Accessed 11 Apr 2016.

[6] Bradshaw SA, Playford ED, Riazi A (2012). Living well in care homes: A systematic review of qualitative studies. Age Ageing.

[7] Jacobs K. RH. Zukunft der Pflegefinanzierung: Eckpunkte für eine tragfähige Reform [Future funding of care: framework for a viable reform]. Das Wissenschaftsforum in Gesundheit und Gesellschaft. 2011;3:14–22.

[8] Theile G, Winter A, Hummers-Pradier E, Junius-Walker U (2012). Das geriatrische Basisassessment in der Hausarztpraxis [The geriatric basic assessment in general practice]. Z Gerontol Geriat.

[9] Deck R, Glaser-Möller N, Kohlmann T (2012). Rehabilitation bei sozial benachteiligten Bevölkerungsgruppen [Rehabilitation of socially disadvantaged population groups].

[10] Kassenärztliche Bundesvereinigung [National Association of Statutory Health Insurance Physicians]. Ambulante geriatrische Komplexbehandlung im therapeutischen Team [Ambulatory Geriatric Rehabilitation at the therapeutic team]. 2007. https://www.aerzteblatt.de/download/files/2007/07/x0000125275.pdf. Accessed 7 Mar 2016.

[11] Nikolaus T, Specht-Leible N, Bach M, Oster P, Schlierf G (1999). A randomized trial of comprehensive geriatric assessment and home intervention in the care of hospitalized patients. Age Ageing.

[12] Meinck M, Freigang K, John B, Keitel C, Puls E, Robra B (2003). Wohnortnahe geriatrische Rehabilitation [Geriatric Rehabilitation close to home]: Evaluation zweier Modelle anhand medizinischer Verlaufskriterien [Evaluation of two models based medical criterias]. Rehabilitation.

[13] MDK - Medizinischer Dienst der Krankenversicherung [Medical Service of the health insurance]. Pflegebegutachtung [nursing care assessment].

[14] Sundmacher L, Fischbach D, Schuettig W, Naumann C, Augustin U, Faisst C (2015). Which hospitalisations are ambulatory care-sensitive, to what degree, and how could the rates be reduced?: Results of a group consensus study in Germany. Health Policy.

[15] Purdy S, Griffin T, Salisbury C, Sharp D (2009). Ambulatory care sensitive conditions: Terminology and disease coding need to be more specific to aid policy makers and clinicians. Public Health.

[16] Stuart EA (2010). Matching methods for causal inference: a review and a look forward. Statist Sci.

[17] Putter H, Fiocco M, Geskus RB. Tutorial in biostatistics: competing risks and multi-state models. Stat Med. 2007;26:2389–430. doi:10.1002/sim.2712. 10.1002/sim.2712 17031868

